# RECOTOX, a French initiative in ecotoxicology-toxicology to monitor, understand and mitigate the ecotoxicological impacts of pollutants in socioagroecosystems

**DOI:** 10.1007/s11356-018-2716-5

**Published:** 2018-07-18

**Authors:** Christian Mougin, Véronique Gouy, Vincent Bretagnolle, Julie Berthou, Patrick Andrieux, Patrick Ansart, Marc Benoit, Michaël Coeurdassier, Irina Comte, Cécile Dagès, Laurence Denaix, Sylvie Dousset, Laure Ducreux, Sabrina Gaba, Daniel Gilbert, Gwenaël Imfeld, Lucie Liger, Jérôme Molénat, Sylvain Payraudeau, Anatja Samouelian, Céline Schott, Gaëlle Tallec, Emma Vivien, Marc Voltz

**Affiliations:** 1 UMR ECOSYS, INRA, AgroParisTech, Université Paris-Saclay, 78026 Versailles, France; 2 UR RiverLy, Irstea, 69626 Villeurbanne, France; 3 UMR 7372 CEBC, CNRS & Université de La Rochelle, 79360 Villiers en Bois, France; 4 UR ASTRO, INRA, 97170 Petit-Bourg, France; 5 UR HYCAR, Irstea, 92761 Antony, France; 6 UR ASTER, INRA, 88500 Mirecourt, France; 7 UMR Chrono-Environnement, Université Bourgogne Franche-Comté, CNRS, INRA, 25000 Besançon, France; 8 CIRAD, 34098 Montpellier, France; 9 UMR LISAH, Univ. Montpellier, INRA, IRD, Montpellier SupAgro, 34060 Montpellier, France; 10 UMR ISPA, INRA, Bordeaux Sciences Agro, 33882 Villenave d’Ornon, France; 11 UMR LIEC, CNRS, Université de Lorraine, 54506 Vandoeuvre les Nancy, France; 12 BRGM, 97170 Petit-Bourg, France; 13 USC 1339, Centre d’Etudes Biologiques de Chizé, INRA, F-79360 Villiers-en-Bois, France; 14 UMR LHyGeS, CNRS, ENGEES, Université de Strasbourg, 67084 Strasbourg, France

**Keywords:** Ecotoxicology, Toxicology, Contamination, Agroecology, Biodiversity, Farming practices, Catchment, Hydrology, Metal, Biopesticides, Biocide, Upscaling, A posteriori risk assessment

## Abstract

RECOTOX is a cross-cutting initiative promoting an integrated research to respond to the challenges of monitoring, understanding, and mitigating environmental and health impacts of pesticides in agroecosystems. The added value of RECOTOX is to develop a common culture around spatial ecotoxicology including the whole chain of pressure-exposure-impact, while strengthening an integrated network of in natura specifically equipped sites. In particular, it promotes transversal approaches at relevant socioecological system scales, to capitalize knowledge, expertise, and ongoing research in ecotoxicology and, to a lesser extent, environmental toxicology. Thus, it will open existing research infrastructures in environmental sciences to research programs in ecotoxicology of pesticides.

## Introduction

One of the greatest challenges for ecosystem sustainability and human health is currently the ever-increasing pollution of all environmental compartments by chemicals of various types and properties. For many years, the scientific community has emphasized the lack of long-term field- and landscape-scale monitoring of the fate of chemicals in water, soil, air, and biota, and of their negative effects on living organisms. AllEnvi, Aviesan, and Athena, the French alliances that combine the national research potential and coordinate the scientific strategy, created the French initiative on toxicology, ecotoxicology, epidemiology, and social sciences, that underlined these points in 2013 (AllEnvi 2013). Because ecotoxicological studies are most often carried out in conditions far from real conditions (Topping et al. 2015), they limit our capacity to identify, predict, and mitigate effects of chemicals (Beketov and Liess 2012). In particular, interactions between processes at the relevant ecological and spatiotemporal scales, and the complex effects of pesticides in mixtures, should be taken in consideration (Pelosi et al. 2017; Shugart 2017). However, the key processes and factors controlling ecosystem exposure, vulnerability, and resilience at appropriate scales often remain elusive (Moe et al. 2013). Contaminant dynamics of contaminants in socioecological systems (SES) such as agroecosystems, which results in multicompartment exposures of organisms with different ecology (e.g., interindividual interactions and social behavior, trophic relationships), are the current bottlenecks that prevent prediction and mitigation of pollutant impacts at landscape or regional scales (EFSA 2010; Topping et al. 2014, 2015).

In this context, pesticides used in agriculture are of specific concern because:

1. Agricultural systems, subject to frequent and diverse pesticide inputs, have been treated for more than 70 years with pesticides and other biocides to increase crop production and quality (Kremen and Miles 2012; Oerke 2006) and remain mostly dependent on their use. It is now recognized that pesticide use has led to the contamination of both environment and food (Carvalho 2017; Mitchell et al. 2017). Their use and dispersion are environmental and societal concerns due to their potential environmental and health impacts, especially on farmers or residents of agricultural areas (INSERM 2013). In addition, the current central status of pesticides in conventional agriculture makes it difficult to reduce their use despite current national and European policies (EU Framework Directive on the sustainable use of pesticides).
2. Both the large range of properties of the active ingredients and coformulants (toxicity, retention, degradation, mobility…) and the variable characteristics of the hydroagroecosystems (e.g., soil, bedrock, hydroclimatic conditions, land use and management, from conventional to organic agrosystems…) where they are applied require the implementation of interdisciplinary research to address the complex issues of ecotoxicology and environmental toxicology of pesticides at the landscape or territorial scale.
3. Institutional and professional organizations request integrated methods and models to improve monitoring, assessment, management, and mitigation of both environmental and human health risks associated with pesticides. Indeed, assessing the impacts of these substances on ecosystems, and their possible consequences on human health, in a context of global change and of exposure to multiple stressors, remains a priority issue for policy makers. Under the French legislation, the “phytopharmacovigilance” concept has been erected in order to monitor the adverse effects of pesticides used in agriculture in relation with the granting of their marketing authorizations (https://www.anses.fr/en/content/phytopharmacovigilance).

In that context, RECOTOX (https://www.recotox.eu/) is a cross-cutting initiative to promote integrated research to respond to the scientific challenges of monitoring, understanding, and mitigating the environmental and health impacts of pesticides and biocides in agricultural landscapes, in agreement with the “Eco Health” approach. Here, we describe the scientific and operational objectives of RECOTOX, based on its experimental site network.

### What is RECOTOX?

RECOTOX is an initiative supported by French research institutes (INRA, Irstea, and CNRS) that promotes a common culture of spatial ecotoxicology of pesticides that considers the whole chain pressure-exposure-impact. RECOTOX accounts for a wide range of agropedoclimatical situations, in terms of soil-bedrock, agronomic, and climatic-hydrologic characteristics, influencing pesticide sources or biotransformation (retention-degradation) and transfer processes. RECOTOX, therefore, explores the panel of responses to provide systemic and generic outcomes.

RECOTOX aims to (i) determine pesticide transfers and mitigation in soil/water/atmosphere, (ii) analyze the effects of pesticides on biodiversity and resilience processes, (iii) understand how farmer practices in interaction with the environmental affect pesticides transfers, and (iv) examine socioeconomic processes such as acceptability of changing practices, costs, or expectations of civil society. Consequently, the scientific community embedded within RECOTOX comprises environmental chemists, hydrogeologists and hydrologists of the critical zone, ecotoxicologists, microbiologists, ecologists and toxicologists, agricultural scientists, and scientists of human, economic, and social sciences. The added value of RECOTOX in terms of skill, knowledge, and tool capitalization in the field of ecotoxicology could be transposed to toxicology, and then promote interdisciplinary and federative research projects, e.g., in the field of phytopharmacovigilance.

RECOTOX develops cross-cutting actions, presented hereafter, within a current network of 10 sites.

1. *Promoting an integrated research in ecotoxicology*. The first and main action of RECOTOX is to incubate research projects, involving the wide range of scientific and technological expertise of its partners, and making use of the complementarity of their observatories and skills. As a consequence, RECOTOX aims to create general knowledge, synthesis of information, and facilitate the emergence of integrated research in spatial ecotoxicology among its sites. It will also rely on the homogenization of reference documents and integration of existing metadata in a common open information system. RECOTOX will strengthen in selected sites’ existing measurements of practices and uses, transfers, exposure, or impacts in the different compartments (soil, water, air, food chain), and at the relevant spatial and temporal scales, establish an integrated approach of the pressure-exposure-impact chain. This integrated approach will benefit from ongoing research programs conducted at the concerned research sites, and/or more comprehensive projects to share methods and models previously developed for different sites of the network.
2. *Upscaling approaches in ecotoxicology from plot to landscape*. One current challenge is to include ecological realism into ecotoxicological studies. A major step is to understand the relationships between chemical releases and mitigation, exposure patterns, and population spatiotemporal dynamics in heterogeneous landscapes, in response to agricultural practices at various spatial and temporal scales. More, there is a lack of more realistic biological indicators in the case of moderate and chronic environmental degradation by pesticides, in particular indicators based on networks of interactions between organisms under the influence of the physicochemical conditions of the environment. Ecotoxicologists, as well as risk assessors, express increasing interest in learning more about both exposure to and effects of chemicals at the landscape scales. RECOTOX will also evaluate the effect of remediation solutions (agricultural systems with low pesticide pressure, implementation of buffer zones such as grassed strips, or artificial wetlands) on the measured or expected impacts at the relevant management scales. Here, observation, experimentation, and modeling will be combined.
3. *From ecotoxicology to toxicology*. RECOTOX aims at developing, in some cases, observational studies to improve the characterization of human exposure and exposome, by collecting environmental samples, data, and information on daily environments of volunteers. Actions may include (i) the identification of pesticide sources; (ii) the selection of sentinel populations, highest-exposed individuals, activities, and behaviors that yield exposures; (iii) the evaluation of the magnitude and frequency of exposure; and (iv) the examination of the exposure–response relationships. Such data are particularly necessary to improve performance of epidemiologic studies and reduce uncertainties in risk assessment. These studies will also enable to share experience with epidemiologists. RECOTOX will also fact to integrate both environmental and human exposure assessments (Ciffroy et al. 2016).
4. *Harmonizing analytical methods and measurements, and sharing equipment, documentary resources, and technological intelligence within the sites*. Gathering efforts and skills of all sites involved in RECOTOX is an essential step to develop common standards and methodologies. Indeed, monitoring, measurement, and experimental protocols are often not standardized due to various historical, logistical, or monitoring constraints. Substantial improvements are thus expected in associating existing sites into a network defined by shared complementary scientific objectives. The emergence of new techniques, new experimental protocols, or new scientific questions will be fostered within the community using RECOTOX. This in turn is expected to enhance the convergence towards the hottest topics or newest techniques, and to accelerate knowledge transfer towards application and societal needs.
5. *Developing a focal point to acquire, store, diffuse, and archive data*. RECOTOX aims at offering resources and software tools to end users for data and metadata access, storage, querying, and formatting from modeling services. End users are multiple: researchers, advisers, farmers, contractors, agricultural teachers, and other stakeholders regarding ecotoxicology and toxicology, which requires specific dedicated services. Collecting and opening data (and metadata) implies organizing the network to develop interoperability of data, metrics, datasets, and methods. An appropriate information system will be devoted to data management from new experiments and also to ensure identification and valorization of acquired data currently managed by different and distributed information systems within each observatory.
6. *Communication, exploitation, and dissemination of results*. RECOTOX aims at structuring the scientific community in ecotoxicology and developing interactions with toxicologists. RECOTOX will favor interdisciplinary research programs and promote facilities for an integrated approach, and thus contribute to increase the research potential on continental ecosystems. The wide range of scientific and technological expertise of RECOTOX partners may also serve as an educational support for training in the fields of toxicological risks ecosystems (ecotoxicology) and human (environmental and food toxicology) health.

### RECOTOX, an added value for existing infrastructures in terms of observation, experimentation, and modeling in the field of long-term ecotoxicology

RECOTOX aims at developing research to investigate the long-term effects of chemical pollution induced by human activities, considering the influence of land use, landscape composition and structure, or changes in agricultural practices. It especially contributes to the development of in natura research in ecotoxicology, and addresses the complex interactions between hydrological, chemical, biological, ecological, and human processes relying on an integrated approach from the plot to the regional scale. RECOTOX could be a key support to existing long-term infrastructures for observation, experimentation, and modeling in ecotoxicology, especially providing them realistic in situ knowledge, tools, and expertise at the relevant spatiotemporal scales of landscape management. Some infrastructures can contribute to strengthening, extending, and supporting RECOTOX. For instance, the research platforms of the LTSER Zones Atelier network (http://www.lter-europe.net/lter-europe/infrastructure/networks/national-networks#lter-france), five of which (the Zones Ateliers, ZA) being involved in RECOTOX, will provide a very strong basis for landscape-scale analyses of pesticide distribution within ecosystems, as well as excellent connections with society and stakeholders in general. The Research Infrastructure OZCAR “Network of Critical Zone Observatories” (http://www.ozcar-ri.org/) is a network of sites of continental surfaces (critical zone) with instrumentation on soil, subsurface, water, and ice dedicated to continuous measurement in support of knowledge and modeling of water, carbon, and associated elemental cycling, and comprises two sites of RECOTOX. Then, RECOTOX can also benefit from the Research Infrastructure “Analysis and Experimentation on Ecosystems - France” (AnaEE-France, https://www.anaee-france.fr/en/). AnaEE-France is an integrated network of the major French experimental, analytical, and modeling platforms dedicated to the biological study of continental ecosystems (aquatic and terrestrial). This infrastructure aims at understanding and predicting ecosystem dynamics under global change. AnaEE-France comprises complementary nodes offering access to the best experimental facilities and associated biological resources and data: the Research Infractructure Ecotrons, and semi-natural experimental platforms to manipulate terrestrial and aquatic ecosystems. AnaEE-France also provides shared instruments and analytical platforms dedicated to environmental (micro-) biology, as well as databases and modeling tools designed to represent ecosystem dynamics in coupling ecological, agronomic, and evolutionary approaches. AnaEE-France is already open to the community of scientists in the field of continental ecotoxicology (Mougin et al. 2015; Clobert et al. 2018).

In addition, RECOTOX would benefit from the Research Infrastructure “Agronomic Resources for Research” (AgroBRC-RARe), and its pillar “environmental resources” (BRC4Env, https://www.brc4env.fr/, Mougin et al. this special issue). Indeed, this infrastructure brings together networks of Biological Resource Centers (BRC) maintaining genetic, genomic, and biological resources produced and characterized by research on domestic animals, crops and model plant species, wild relatives of domestic species, microorganisms relevant for agriculture and macrofauna of the environment, and environmental samples. RECOTOX can particularly benefit from its capacity to maintain a large diversity of well-documented resources, to collect novel resources, to contribute to their characterization, to distribute them, and to manage the related data. RARe can also help the sites of the network in strengthening and organizing their ability to store and secure collected samples and their related data in the long-term.

Finally, a scientific animation will be developed in partnership with the network of terrestrial and aquatic ecotoxicology ECOTOX (https://www6.inra.fr/ecotox_eng/). Indeed, ECOTOX has three main objectives: (i) to encourage scientific thinking within the community of ecotoxicologists, whether they are dealing with aquatic or terrestrial ecosystems; (ii) to contribute to defining a national research strategy in this field; and (iii) to favor scientific production (Mougin et al. 2016). For many years, the network has been offering opportunities for exchanges within the French community of ecotoxicologists, especially through regular scientific seminars. RECOTOX is also in line with EcotoxicoMic (https://ecotoxicomic.org/), the international network on Microbial Ecotoxicology (Ghiglione et al. 2016).

### RECOTOX, a network of sites

RECOTOX focuses its activities in a network of instrumented sites mostly belonging to recognized Research Infrastructures (LTSER France, Network of Critical Zone Observatories…), managed by research institutes and universities. The sites cover a variety of soil, agronomic, climatic, and socioeconomic situations, which are reflected in the diversity of agricultural practices, land uses, and landscapes, as well as hydrological and biogeochemical conditions. Their main characteristics are presented in Table 1. RECOTOX addresses research questions at different and complementary spatial scales:

- The plot (or set of plots) which includes sites centered on the design of agricultural systems, allowing reduced agricultural pressure (including pesticides) for the characterization of the determinants and effects of agricultural practices at the local scale,
- The catchment, landscape, or territory scale, which allows the observation, experimentation, and modeling of various agricultural practices, pollutant dissipation/retention/transformation schemes in both cultivated or non-cultivated landscape components, or various exposures and impacts on continental terrestrial and aquatic ecosystems,
- The global (national) scale, where it is intended to organize and cross observations and experimentation results obtained on a similar way in several complementary sites.

**Table 1:** Main characteristics of the sites actually involved in the RECOTOX initiative

Site	Supporting organization	Keywords	Contact
ZAPVS	LTSER-ZA	Plaine et Val de Sèvre Field crops, landscape Transfer, exposure, impact, modeling weeds, bees, birds, citizens	V. Bretagnolle, S. Gaba, UMR CEBC
ZAS-ORACLE/BVRE Orgeval	LTSER-ZA and OZCAR	Paris basin context Field crops Hydrology, transfer, drainage, constructed wet lands, fish exposure, impact	G. Tallec, P. Ansart, UR HYCAR
ZAM	LTSER-ZA	Lorraine context Field crops, drainage, constructed ponds or ditches, constructed wetlands	S. Dousset, UMR LIEC; M. Benoit, UR ASTER
ZABR-Ardières	LTSER-ZA	Beaujolais context vineyards, landscape Transfer, exposure, impact, modeling, buffer zones, management, soil, and aquatic microbial communities Aquatic macroinvertebrates	V. Gouy, L. Liger, UR RiverLy
ZAAJ	LTSER-ZA	Jura, low mountains context Rivers; grassland, caves, mixed farming breeding landscape, exposure, impact rodents, birds, bats, wildlife (fox, hare, etc.), aquatic invertebrates	M. Coeurdassier, D. Gilbert, UMR Chrono-Environnement
OMERE	OZCAR	Mediterranean context Vineyard (Roujan), cereals, pastures (Kamesh) intermittent flow, buffer zones, soil management	C. Dagès, J. Molénat, A. Samouelian, M. Voltz, UMR LISAH
OPALE	PNAC	Tropical context, West Indies banana plantations, grasslands transfer, multiexposure	P. Andrieux, UR ASTRO; I. Conte, CIRAD; L. Ducreux, BRGM
OBSERVOX		Crops and vineyard Contamination, practices, transfer	M. Benoit, UR ASTER
Hohrain-Rouffach		Alsatian Piedmont context vineyard transfer, grassed interrows, constructed wetland	G. Imfeld, S. Payraudeau, UMR LHyGeS
Quasaprove		Various French contexts Agricultural practices Field crops Contamination, exposure, soil-plant-transfer	L. Denaix, E. Vivien, UMR ISPA

*LTSER-ZA* Long Term Socio-economic and Ecosystem Research - Zones Ateliers; *OZCAR* French network of Critical Zone Observatories; *PNAC* French “Plan National d’Action Chlordécone”

The sites are mainly concerned with arable crops and wine growing. A few sites also comprise grasslands or tropical crops (Fig. 1). They are described hereafter:

1. The Zone Atelier Plaine & Val de Sèvre (ZAPVS) (http://www.zaplainevaldesevre.fr/, Bretagnolle et al. 2018) is a long-term socioecological research platform where interdisciplinary research interact with stakeholders. The ZAPVS is located south of Niort in “Deux Sèvres” department, Western France, and is managed by the Center of Biological Studies of Chizé (CEBC), UMR CNRS, and University of La Rochelle. Since 1994, the Zone Atelier has set up programs using long-term monitoring and in situ experiments involving local citizens or farmers, to address key socioeconomic challenges such as how farming practices (including pesticide applications) affect biodiversity and ecosystem services. The ZAPVS covers 450 km^2^, including the forest of Chizé, and is a typical rural area with a temperate Atlantic oceanic climate. It includes around 450 farms with high diversity of agricultural strategies with organic farming, conservation farming, and conventional farming. Since 2003, half of the area is designated as a Special Protection Area (NATURA 2000 network) for conservation of remarkable or endangered bird species such as the little Bustard *Tetrax tetrax* or harriers *Circus* spp. From 1994 onwards, biodiversity surveys have been carried out every year in 120 to 200 farmers’ fields. Monitoring started with birds and small mammals in 1994, ground-dwelling arthropods since 1995 and then other arthropods (including bees, hoverflies, grasshoppers, and spiders), plants since 2005, and soil organisms since 2016. In addition to biodiversity monitoring, farming practices have been monitored by means of farmer interviews (about 150 fields per year) which allow to quantify pesticide uses and analyze the relationships between this use and biodiversity. The ZAPVS also hosts the ECOBEE platform (Odoux et al. 2014) which monitored honey production and honey bee population dynamics since 2008. Using this platform, studies demonstrated, for the first time, the effect of neonicotinoid pesticides (thiamethoxam used for oilseed rape in France, Henry et al. (2012) and imidachloprid Henry et al. (2015)) on the survival of honey bee.

**Fig. 1:**
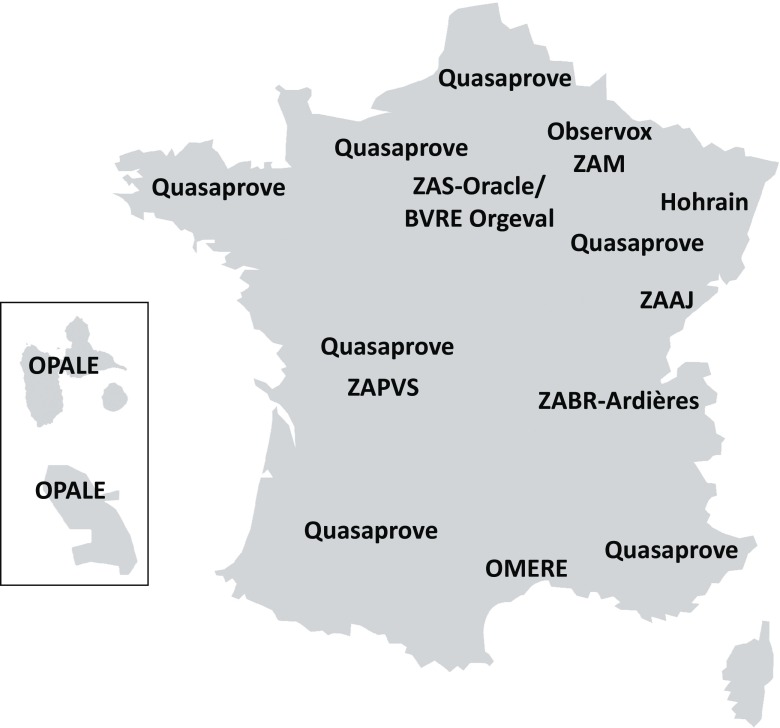
Map of the location of the French sites currently included in the RECOTOX network
2. The Zone Atelier Seine (ZAS**)** comprises the observatory ORACLE/BVRE Orgeval (http://gisoracle.irstea.fr). Located 70 km east of Paris, the Orgeval catchment supports research on understanding the processes and developing integrative modeling of the hydrological functioning of its small upstream catchment. Started in 1962, the Orgeval catchment is managed by Irstea. This site belongs to the ORACLE observatory (1800 km^2^) which is composed of the Grand Morin and Petit Morin catchments. The ORACLE observatory gathers many national and international research units in the GIS ORACLE (Group of Scientific Interest) created in 2011. The Orgeval catchment is also part of the “Zone Atelier Seine” thanks to the Interdisciplinary Research Program in Environment on the Seine basin (Piren Seine) and it is also part of the IR OZCAR. The ORACLE observatory is dedicated to the improvement of the knowledge on the hydrological and biogeochemical functioning of sedimentary basins in rural anthropized systems. Its objective is the observation and modeling of streamflow and of water and pollutant transfer processes at various scales. It also works on the optimization of data acquisition methods. ORACLE, with its set of nested subbasins and its perennial long-term observations (more than 50 years), allows to address the scaling and global change issues. The Orgeval catchment area covers 104 km^2^. It is mostly constituted of drained fields and devoted to field crops. It has a direct influence, in terms of quantity and quality, on the flows going from the Marne to the Seine Rivers until the Paris conurbation. On the Orgeval catchment, chronicles of streamflow and water quality data are available since 1962 and 1978, respectively, and the monitoring of pesticides started in 2008, in the context of two projects: Phyt’ORACLE (EC2CO) and PIREN Seine (Blanchoud et al. 2010; Tournebize et al. 2013). The interest of ecotechnologies (as constructed wetlands) to reduce pesticide transfer is also studied.
3. The Zone Atelier Moselle (ZAM) (http://zam.univ-lorraine.fr/) is an environmental research platform created in 2000 and located in the Grand Est region. Currently, the ZAM gathers a Lorraine scientific community (CNRS, INRA, ANSES, and Université de Lorraine) around the protection of water resource of the Moselle River. The ZAM covers 15,400 km^2^ in France and presents a highly contrasted occupation of the territory combined with specific anthropic pressures. The upper part of the basin is dominated by the Vosges Mountains, sitting on acidic soils (crystalline or sandy substrates). The middle and lower parts of the French basin correspond to a succession of cuestas characterized by limestone plateaus with permeable soils and plains with a dominant clay soil. Agriculture, especially mixed farming and livestock farming, is present on the middle part of the basin, which receives regular agricultural inputs (fertilizers, pesticides, etc.). The downstream part of the basin is characterized by increasing urbanization (Nancy, Toul, Metz, Thionville) and by the heritage of Lorraine’s industrial past (iron mining and steel industry). Still, throughout its journey, the Moselle River contributes to maintain wetlands areas with a rich biodiversity (regional nature reserve of the Moselle Sauvage, for example). The ZAM research actions are structured around five main disruptions linked to geomorphology, land use, and human activities: the acidification of streams and rivers in the Vosges mountains; pesticides and their residues, mainly linked to agricultural activities (Lazartigues et al. 2013; Vallée et al. 2016); metals and persistent organic pollutants from industrial activities; emerging domestic pollutants from urban areas; microbiologic disruptions. Particular attention is paid to the development of remediation solutions. Because of its cross-border nature, the Moselle catchment raises issues that extend beyond the national territory to its two neighboring countries, Germany and Luxembourg.
4. The Zone Atelier Bassin du Rhône (ZABR) (http://www.zabr.org/) comprises the Ardières-Morcille experimental site (https://saam.irstea.fr/), which studies the impacts of anthropization, especially wine growing, on river water quality and aquatic ecosystem functioning in order to highlight the key involved processes, biological adaptations, and remediation trajectories. The Ardières-Morcille experimental site belongs to the Rhône Basin Long-term Environmental Research Observatory (ZABR) which comprises a set of observatories and experimental sites (e.g., SIPIBEL, Mountain Lakes observatory…) devoted to a better understanding and management of the Rhône River basin. The Ardières-Morcille experimental site was set up in 1987 by the Cemagref, the regional service for water use and supply and the Beaujolais Development Committee. It moved in 2007 to the ZABR (which has been labeled SOERE ZA in 2001). Among the other experimental sites and observatories of the ZABR, the Ardières-Morcille experimental site was chosen in priority to be part of the RECOTOX network because of the anteriority and history of the research devoted to the fate and effects of pesticides used in agriculture, and because of the interdisciplinary approach developed along the fate-exposure-impact chain. Researches on the Ardières-Morcille experimental site also contribute to providing operational tools to better understand and implement buffer zones in catchments so as to limit the diffuse agricultural pollutions of rivers (outputs in terms of results and expertise especially support the Buffer Zone Working group created in 2011 on the ONEMA initiative). The Ardières-Morcille experimental site consists of two nested catchments: the Morcille catchment (8 km^2^) and the Ardières catchment (150 km^2^). Located northwest of Lyon in continental climate, the Ardières-Morcille experimental site is characterized by intensive wine growing. It is representative of the agricultural, soil, and climatic conditions of the central and northern Beaujolais. The first studies (since 1987) started on the Morcille subcatchment which is now equipped with long-term hydrometeorological stations and experimental subsites. The recent studies on the Ardières catchment (since 2011) enable both to address the question of hydrological processes and modeling scaling up and to strengthen the involvement of the economic and social sciences. Four research themes are crossed on the Ardières-Morcille site: observation and modeling of hydric transfers of pesticides; characterization of the chemical (organic, metal…) contamination of the river; development of knowledge and indicators of the in situ impacts of pesticides on aquatic ecosystems and their recovery capacity (resilience); development of methods and tools to help public decision-makers to understand and use the research results (Rabiet et al. 2010; Montuelle et al. 2010; Gatel et al. 2016). The coexistence of both scientific questions and socioeconomic issues on the Ardières-Morcille experimental site is one of the originality of this ZABR site, as well as the interaction between physical, chemical, biological, social, and human sciences. For example, it studies the “resistance” of local stakeholders to changes.
5. The Zone Atelier Arc Jurassien (ZAAJ) (http://zaaj.univ-fcomte.fr/) is an area of 13,500 km^2^ that covers a large part of the Jura medium mountains in Bourgogne-Franche-Comté and Rhône-Alpes regions, eastern France. It is composed of grasslands exploited for cheese production, karst and rivers, forests, peatlands and wetlands, and urban areas. ZAAJ observatories are designed to promote long-term interdisciplinary research on the dynamics of the socioecosystems of this territory. They contribute to capitalize on field data and to analyze and promote the results from long-term observation schemes at the interface between ecology, environment, and society. The main researches questioned the impacts of human activities, including those due to environmental contaminants, and their implications in terms of ecosystems health, social economic issues, and biodiversity conservation. They aim to assess the vulnerability of ecosystems and population at risk and to provide predictive and management tools to help maintain ecosystem services and protect population and ecosystems’ health. Among questions related to RECOTOX, we can cite for instance vole outbreak management in grassland agrosystems and side effects on wildlife (Coeurdassier et al. 2014), contamination of the Loue River by pesticides, other toxicants and nutrients, and impacts on aquatic ecosystems (Chiffre et al. 2016), or exposure of chiroptera to contaminants (Afonso et al. 2016). The ZAAJ benefits from more than 25 years of experience and monitoring (e.g., vole population dynamics, predator-prey relationships, pesticide use…) and was accredited by the CNRS Ecology and Environment Institute (INEE) in 2013. It belongs now to the national experimental site network, which is part of the national LTER network and federates a partnership network of eight research teams, and stakeholders such as institutions, farmers and technical institutes, conservationists, and game federations.
6. The agro-hydrological observatory OMERE (Observatoire Méditerranéen de l’Environnement Rural et de l’Eau, INRA/IRD, http://www.obs-omere.org) is composed of two cultivated headwater Mediterranean catchments: the Roujan catchment (0.91km^2^) located in France (Languedoc region) and the Kamech catchment (2.63 km^2^) located in Tunisia (Cap Bon Peninsula). The OMERE observatory was set up in 2002 to understand the impact of agricultural and land management on mass fluxes in typical farmed headwater catchments of the Mediterranean region. Within this main objective, the OMERE aims at studying the natural and anthropogenic drivers of pesticide contamination of soil and water across scales from plot to catchment scales. The observatory helps to address the effect on pesticide transfer of highly variable hydroclimatic conditions characterizing Mediterranean regions (long drought periods, intense storms), as well of soil type, crop treatment, and land management. The two catchments are similar with respect to environmental and climatic conditions. They differ according to the change in land use and human activities they are submitted to. In the Kamech catchment, cereals, legumes, and irrigated market gardening are mainly cultivated and a progressive intensification of agriculture occurs with a full use of the area available for agriculture and an increasing application of fertilizers and pesticides. In the Roujan catchment characterized by wine growing, intensification of agriculture has already been operated for a few decades and has led to the creation of a network of ditches and human-made slopes. So far, the dynamics of pesticides, mainly herbicides, has been studied in Roujan catchment. Pesticides have been monitored in soil, runoff water, groundwater, and rainfall. In surface waters, the monitoring is performed from plot to catchment scale using a nested network of hydrometric stations. The monitoring design is based on the knowledge of the applied treatments that are also recorded.
7. The observatory OPALE (Inra/Cirad/BRGM/IRD, http://obs-opale.org) is an observatory of agricultural pollution in the French West Indies (tropical conditions) composed of two equipped catchments: one in Guadeloupe and the other one in Martinique. It is dedicated to the study of the fate of pesticides (including chlordecone) in the environment according to agricultural practices. The OPALE observatory (Andrieux et al. 2014) is composed of two study areas: the Rivers Pérou-Pères catchment (25 km^2^) in Capesterre-Belle-Eau in Guadeloupe, and the Galion catchment (40 km^2^) in Martinique. Previously called OPA-C, the OPALE observatory was accepted in the frame of the call for projects of the Chlordecone National Action Plan—catchment (PNAC-BV) in May 2012. This project, lasting from 2012 to 2014 enabled the implementation of equipment in the two catchments thanks to a partnership between the BRGM, CIRAD, IRD, and INRA. The Pérou-Pères River catchment in Guadeloupe is located on the edge of the volcano *la Souffrière*. It is composed of ashy soil on a recent geological formation. The Galion catchment in Martinique is located on well-developed soils with old geological formation. Those two sites are typical of tropical conditions with the leaching of soils with a high organic matter content. They enable to cover the main crops specifically of the French West Indies: sugar cane and banana. Therefore, those two sites have been affected by a heavy use of chlordecone between 1972 and 1993. The OPALE observatory aims at monitoring the agricultural practices and surface or groundwater contamination on a small agricultural area (Crabit et al. 2016). Focusing initially on chlordecone, the observatory then broadened its study to all the French West Indies ecosystem pollutions (Fernández-Bayo et al. 2013; Mottes et al. 2017). Beyond the data collection and production, the observatory aims to share and pass data on to the scientific community and a broader public.
8. The observatory OBSERVOX (Siabave/Univ Reims/Inra (https://www6.nancy.inra.fr/sad-aster/Projets/Observox) is an agroenvironmental observatory on the Vesle catchment. It aims to guide territory stakeholders in the coconstruction of a collective knowledge management device about agricultural practices, in order to help the implementation of actions to improve the water resource quality. OBSERVOX is a territorial observatory of agricultural practices (especially plant protection practices) on the Vesle catchment up to the Couraux water catchment area, which covers 700 km^2^. It was created in 2007 in the frame of the AQUAL objective contract. OBSERVOX consists of five subcatchments. It is carried by the SIABAVE (Intercommunal Vesle Catchment Management Syndicate) and the scientific part is led by the CReSTIC (Research Center in Sciences and Technology of Information and Communication – Université Reims Champagne Ardennes) and the ASTER unity of INRA of Mirecourt. The Seine Normandie Water Agency and the FEDER take part in the cofinancing of the project. In OBSERVOX, two sites are part of the RECOTOX network: the site of Cernay-les-Reims and the site of Sommevesle. The first one covers 37 ha of vineyards localized on the Reims and Berru mountain slope. Approximately 50 farmers work on this site. The second one covers 830 ha of croplands. Those two sites are characterized by a temperate continental climate tending towards oceanic and chalk soils of the Champagne region. This territory is typical of the Champagne region. The OBSERVOX observatory aims to increase knowledge in partnership with territory stakeholders on water resource quality and changing agricultural and wine-making practices, as well as on the factors affecting molecule transfers towards water resource. It has three objectives: access the change in pesticide pressures linked to their use in vineyards and croplands; explain this evolution; link this evolution to analyzed pesticides and metabolites in waters from the Couraux water catchment area. Benefiting from a 10-year monitoring in the Champagne region, the OBSERVOX site is also located in an agricultural area where there is a heavy use of pesticides. Therefore, this site is particularly relevant to study the impact of pesticide use on water resources.
9. The Hohrain-Rouffach vineyard (Alsace, https://www.lhyges.unistra.fr/ROUFFACH,460) enables the quantitative evaluation of matter transfer processes (water, solids, solutes, especially pesticides) in relation to hydrological and agronomic forcing and human activities in vineyards in order to understand, quantify, and predict the hydrological and biogeochemical functioning of an agrosystem in perennial culture. Located 15 km southwest of the town of Colmar in Alsace, the site is one of the observatories of the Laboratory of Hydrology and Geochemistry of Strasbourg (LHyGeS, UMR7517 CNRS/UNITRA/ENGEES). A 43-ha vineyard catchment was equipped in 2003 for the monitoring of pesticides, and then in 2009 for the monitoring of flows and cycles of solutes, including pesticides and metals (Cu, Zn, etc.) and integrating an artificial wet buffer zone at the outlet of the 43-ha catchment. The Hohrain’s catchment team is composed of researchers of the LHyGeS and lecturers of ENGEES (Ecole Nationale du Génie de l’Eau et de l’Environnement) and EPLEFPA (College for winegrowers), as well as the city of Rouffach and winegrowers in the catchment area. The monitoring of the Hohrain-Rouffach catchment has been developed in the framework of the LIFE Environment project ArtWET (2006–2011), the INTERREG IV PhytoRET (2011–2014), and the PACOV project (2014–2018, AERM), coordinated by the LHyGeS. The Hohrain-Rouffach site is representative of agriculture, soil (calcareous loess), and continental climatic conditions of the Alsatian Piedmont. The site is equipped to quantify solute flows at the catchment scale and the vineyard plot (two 1341-m^2^ plots equipped). The artificial stormwater wetland zone (320 m^2^ and 1500 m^3^) located at the catchment’s outlet is studied since 2009 in terms of reactive transport of pesticides and metals at the water/plant/sediment interface. For 10 years, the catchment has served to collect information on water and chemical flows as well as land use data. A specificity of the catchment is that it enables to investigate the plot–catchment–wetland buffer zone continuum (Imfeld et al. 2013). Among other things, it also enables to study the phytoremediation and organic (Maillard and Imfeld 2014) and inorganic (copper and zinc, Babcsányi et al. 2016) pesticide degradation potential of the stormwater wetland.
10. The QUASAPROVE network (http://www.quasaprove.org/moodle/) is an observatory devoted to the study of diffuse contamination of crop fields by mycotoxins, trace elements, and pesticide residues. It is an agronomic tool created in 2010 in the frame of the Combined Technology Network (CNT) in order to focus on the thematic field of sanitary quality of major agricultural products. Quasaprove is a network of plots whose agropedogeochemical conditions are well known. It enables to perform full-scale tests on hypotheses and models about the sanitary quality of crops in pre- and post-harvest (cereals and oilseeds). The Quasaprove network is spread on the whole French territory. It is located in seven INRA sites, two Terres Inovia stations, four ITAB stations, 11 agricultural secondary schools sites, and one Arvalis site in Boigneville. The seven involved INRA sites are as follows: UE Domaine expérimental de la Motte au Vicomte de Rennes, UE 0802 d’Agronomie de Toulouse, UE 1246 Grandes Cultures Versailles-Grignon, UE 1373 Fourrages Environnement Ruminants de Lusignan, UMR EMMAH Domaine St Paul Avignon, UE d’Epoisses, UE Grandes Cultures Innovation Environnement – Picardie. Quasaprove is led by ACTA and INRA. In the Quasaprove network, seven sites belong to the RECOTOX network. Located in Auzeville in Haute-Garonne, Lusignan in the Vienne, Epoisses in Côte-d’Or, Le Rheu in Ile et Vilaine, Versailles in the Yvelines, Estrées-Mons in the Somme, and Avignon in the Vaucluse; those sites cover a wide range of soil (calcosols, calcisols, rendzina, luvisols, etc.) agricultural practices and climate conditions all over the French territory. Conventional and organic croplands (common wheat, durum wheat, and sunflower) are prevailing in the studied sites. These focus on diffuse agricultural contamination in different compartments (soil, plant) and try to understand the sources and accumulation pathway of pollutants. The CNT Quasaprove activities deal with scientific and technical questions in relation with the source, fate, and management of contaminants (mycotoxins, trace elements, organic or inorganic emerging contaminants, pesticide residues, etc.) and non-desirable organisms which jeopardize the sanitary quality at every stage of the food chain. The objectives of the CNT Quasaprove are to assess the contamination levels of crops and agricultural soils; quantify the plot trace elements flow and assess the impact of practices on soil contamination; test characterization tools of the trace element availability, test at a field scale the hypotheses and predictive models of the crop contamination risk; keep a sample bank for further analysis on contaminants not yet studied. The Quasaprove network benefits from a chronicle of the exogenous intakes (fertilizers and pesticides). The multicontamination approach is studied in priority. One of its originality is also to take simultaneously into account the soil and plant compartments with the subsequent difficulty to analyze and model transfers.

### Examples of research projects hosted by RECOTOX sites

Several sites already host research projects in the context of the chain pressure-exposure-impact applied to pesticides. The RECOTOX network offers the perspective to extend them and to address new issues. Examples of projects are presented hereafter:

1. *Development of in situ tools and indicators to better assess and manage the relationship between pressures and impacts of pesticides in surface water*. Grab sampling may be scarcely adequate to evaluate the impact on surface waters of pesticides used in agriculture because of its poor spatiotemporal representativeness compared to the high variability of the contamination patterns. In addition, pesticide concentrations need to be supplemented with biological indicators to assess their actual impact on ecosystems. The objectives of the Ecophyto-1 project are to validate both chemical and biological integrative tools applied together in the main river of the ZABR-Ardières site, so as to identify the coherence and complementarity of their responses according to an upstream-downstream gradient. The projects are supported by the French national program Ecophyto and the French National Office of Water and Aquatic Environments on the ZABR-Ardières site. The ZABR-Ardières basin is of interest to reach this goal: (i) its quasi monoculture of vines and its relatively homogeneous soils make it possible to select comparative measurement stations; (ii) agricultural practices, moderate to high slopes, and climate characteristics are favorable to pesticide transfer to surface water; and (iii) the wide range of pesticide substances (herbicides, fungicides, insecticides) used for vine protection allows exploration of different physicochemical properties and associated pesticide fate. The tested chemical tools were passive samplers as, for example, Silicone Rods (SR) dedicated to hydrophobic to moderately hydrophilic compounds. In total, 28 pesticides (14 herbicides, 6 fungicides, and 6 insecticides) were analyzed as well as potential confusing parameters (metal(oïd)s, nutrients, temperature). In parallel, the biological approach consisted in an in situ study of a range of selected impacts on microbial and invertebrate communities. As an example, the following were measured: (i) leaf litter decomposition, which is mediated by the combined action of natural microbial and invertebrate communities; (ii) fungal biomass within leaf-associated natural microbial communities; (iii) alimentation and acetylcholine esterase inhibition in standardized caged Gammares (*Gammarus fossarum*). The tools have been applied during June 2014 and June 2015 (corresponding to a period of pesticide application on vineyard) in the Ardières River at three measurement stations from upstream to downstream. The results (Le Dréau et al. 2015, 2016) showed the high coherence of chemical and biological tool responses. All of them were able to reflect the spatial contamination gradients as well as the specific contamination and impact associated. Thus, decomposition rates and fungal biomass decreased from up- to downstream, in relation to the observed increase in fungicides and organophosphorous insecticides concentrations, the latter also being consistent with the acetylcholine esterase inhibition in the caged Gammares. Biological impact was observed during the two studied experimental years, while pesticide contamination decreased in 2015 compared to 2014. Finally, their sensitivity, reactivity, and integration capacity make these tools relevant and promising to assess agricultural pesticide impact on surface water in addition to grab sampling.
2. *Investigating pesticide transfer and impact on biodiversity and its ecological functions at the landscape scale*. Agricultural landscapes are spatially heterogeneous because of the variety of cultivated land cover types that are distributed in a complex spatial pattern and interspersed with semi-natural and/or uncultivated habitats like woodlands, hedgerows, field margins, or permanent grasslands. These landscapes are also highly heterogeneous in time with a diversity of crop sequences and with within-year management practices (e.g., timing of pesticides applications). Landscape organization can modulate the resistance and resilience of ecosystems to the impacts of anthropogenic disturbances such as soil pollution, particularly by pesticides. Moreover, landscape elements influence the distribution of contaminants, exposure of living organisms, ecological processes related to ecosystem services, and therefore agroecosystem functioning. For instance, the presence of hedges may limit pesticide transfer or a high percentage of organic farming or grassland in a landscape may buffer the negative effect of pesticides. Unraveling the respective influence of landscape organization and pesticide inputs can thus provide recommendations for risk assessors and suggest spatial arrangement that best support multifunctional and resilient agricultural landscapes. However, assessing the pesticide transfer and risk at landscape scale is challenging and requires dedicated infrastructures. The extensive knowledge of land use in the LTSER Zone Atelier Plaine & Val de Sèvre (ZA PVS) as well as available data on farming practices in the area allows investigating how local farming practices and landscape organization affect biodiversity, ecosystem functions, and pesticide transfer. Three projects address this issue in the LTSER ZA PVS: BIOSERV (A multiservice analysis of the effect of organic farming at the landscape scale), funded by INRA metaprogram ECOSERV, test this hypothesis that the flows of goods and services that organic farming will benefit crop production and farmers profitability both in organic farming and conventional farming fields through higher regulation services; RESCAPE (RESistance of agricultural landSCAPEs to pesticide transfers in soils and living organisms) funded by French Biodiversity Agency on “Resistance and Pesticides” research call, with the fees for diffuse pollution coming from the Ecophyto Plan; and PING (Agricultural Practices, INteractions with the landscape, and wildlife exposure to Glyphosate: an ecotoxicological and socioeconomic approach) funded by Metaprogramme SMaCH of INRA aims to assess the impact of landscape features (composition and configuration) and agricultural practices (especially pesticide inputs) on pesticide transfers in soils and living organisms. All projects use an integrated and interdisciplinary approach. BIOSERV analyzes the effect of landscape composition (percentage of organic farming, conventional farming, and semi-natural habitat) and their spatial arrangement (i.e., aggregated versus evenly distributed) on biodiversity, key ecosystem services (crop production, pollination, biological control, soil fertility, and pesticide degradation), and farmers’ incomes. RESCAPE and PING establish quantitative links between agricultural management, the spatial distribution of pesticides and the effects of pesticides. The PING project also investigates the link between ecotoxicology and socioeconomy by analyzing the farmers’ decision to use glyphosate and how, based on scientific results (i.e., the presence of pesticides in the different compartments of the environment), their perception and use of glyphosate may change. In the three projects, several measurements are performed in arable fields (120 fields) and semi-natural elements (40 to 60; only for RESCAPE and PING) such as soil sampling for physicochemical characterization and pesticide concentrations, biodiversity monitoring (earthworms, carabid beetles, pollinators, and small mammals, some of which are used for pesticide dosing), and ecological functions (pollination, biological control, and crop production in BIOSERV). In RESCAPE and PING, the spatial distribution of pesticides in soils is also studied through the development of analytical methods (Daniele et al. 2018) and lateral transfer models involving atmospheric dispersion. The results on pesticide concentrations in soils and non-target living organisms will allow providing recommendations for risk assessors to increase the resistance and resilience of ecosystems to the impacts of pollution.

## Conclusions

The initiative RECOTOX federates in natura sites that develop a common culture in spatial ecotoxicology in the context of the whole chain pressure-exposure-impact applied to pesticides. RECOTOX will provide an added value to existing research infrastructures for environmental research by launching transversal approaches at relevant socioecological system scales, by capitalizing their knowledge and expertise, and seeking complementarity in the fields of ecotoxicology, and to a lesser extent, in environmental toxicology. RECOTOX will generate proposal and partnership that can serve as a framework for project preparation addressed to funding agencies. Finally, RECOTOX is positioned as the French part of a European network to develop for the research on ecotoxicological effects of pesticides.
